# Simultaneous Determination of Chlorothalonil and 4-Hydroxy-Chlorothalonil in Sulfur-Rich Vegetables by UHPLC-MS/MS with a Synergistic Enzyme Inhibition Strategy

**DOI:** 10.3390/foods14132153

**Published:** 2025-06-20

**Authors:** Fengen Wang, Min Ding, Chao Zhang, Ruiju Li, Cuihua Ma, Xia Li, Zengmei Li, Huidong Li, Hong Zhang, Mengmeng Yan, Ligang Deng

**Affiliations:** 1 Institute of Quality Standard and Testing Technology for Agro-Products, Shandong Academy of Agricultural Sciences, Jinan 250100, China; wfe8520382@163.com (F.W.); dingminzhen@163.com (M.D.); ytzc1212@163.com (C.Z.); liruiju-98@163.com (R.L.); lisa-fsd@163.com (X.L.); lizengmei78@163.com (Z.L.); lihuidong8066@163.com (H.L.); zhanghong7274@163.com (H.Z.); ynky202@163.com (M.Y.); 2 Boxing County Comprehensive Inspection and Testing Center, Binzhou 256500, China; 15254326569@163.com

**Keywords:** chlorothalonil, 4-hydroxy-chlorothalonil, sulfur-rich vegetables, UHPLC-MS/MS, enzyme inhibition

## Abstract

Chlorothalonil and its toxic metabolite, 4-hydroxy-chlorothalonil, pose significant environmental and health risks. However, their simultaneous and accurate detection remains challenging due to their differing ionization efficiencies in mass spectrometry and the interference caused by enzymatic reactions in sulfur-rich vegetables. This study developed a UHPLC-MS/MS method for simultaneous detection of chlorothalonil and 4-hydroxy-chlorothalonil, using an atmospheric pressure chemical ionization (APCI) source, optimizing the probe temperature to 600 °C and a set of optimal chromatography parameters. A low-temperature and acidification synergistic enzyme inhibition strategy was developed, involving refrigerating samples and extraction reagents, acidifying with citric acid before sample homogenization, and extracting with formic acid/acetonitrile, significantly improving chlorothalonil recovery. Method validation demonstrated limits of detection (LOD) and quantification (LOQ) of 0.003 mg/kg and 0.01 mg/kg, respectively, with recoveries of 76.5–91.1% for chlorothalonil and 87.6–96.7% for 4-hydroxy-chlorothalonil. The method was successfully applied in monitoring the residue risks in sulfur-rich vegetables.

## 1. Introduction

Chlorothalonil (2,4,5,6-tetrachlorobenzenedicarbonitrile) is a broad-spectrum contact organochlorine fungicide, which is recognized as one of the top ten commercially significant pesticides worldwide. Its fungicidal efficacy arises from the inhibition of glyceraldehyde-3-phosphate dehydrogenase in fungal cells, rendering it particularly effective against diseases such as downy mildew and soft rot in vegetable and fruit crops [1]. However, increasing attention has been directed toward the environmental and health implications of chlorothalonil residues and its primary metabolite, 4-hydroxy-chlorothalonil (2,4,5-trichloro-6-hydroxy-isophthalonitrile). The International Agency for Research on Cancer (IARC) classified chlorothalonil as a Group 2B potential human carcinogen in 2020, with documented adverse effects including gastrointestinal irritation, hepatotoxicity, and dermal inflammation [2]. Of greater concern, 4-hydroxy-chlorothalonil exhibits significantly higher acute toxicity and environmental mobility compared to chlorothalonil, as indicated by its lower Log*p* value (1.1 vs. 2.9) and increased water solubility [3,4]. These characteristics facilitate its migration into aquatic ecosystems via surface runoff, thereby posing substantial ecological risks [5,6,7]. In response to these concerns, the European Union implemented a comprehensive ban on chlorothalonil in 2019 [8], which has spurred global initiatives to strengthen residue monitoring protocols. The Codex Alimentarius Commission (CAC) stipulates a maximum residue limit (MRL) of 5 mg/kg for chlorothalonil in flowerhead brassicas (including broccoli, Chinese broccoli, and cauliflower). The European Union sets an MRL of 0.01 mg/kg for chlorothalonil in vegetables ((EU) 2021/155), while China establishes an MRL of 5 mg/kg for chlorothalonil in Brassica vegetables (GB 2763-2021 [9]).

Analytical methodologies for the detection of chlorothalonil and 4-hydroxy-chlorothalonil primarily rely on gas chromatography (GC), gas chromatography/mass spectrometry (GC-MS), and liquid chromatography/mass spectrometry (LC-MS) techniques [10,11]. Although GC-based methods have become mainstream detection methods due to their high sensitivity and stable performance [12,13], these techniques are limited in their ability to achieve simultaneous detection of 4-hydroxy-chlorothalonil due to its strong polarity and high boiling point. In contrast, LC-MS/MS offers distinct advantages for the determination of moderate polar compounds. In negative ion mode using electrospray ionization (ESI), 4-hydroxy-chlorothalonil demonstrates high ionization efficiency due to the presence of hydroxyl polar groups. However, ESI is less effective for ionizing weakly polar chlorothalonil, whose molecular structure (containing a benzene ring with four chlorine atoms and two cyano groups) exhibits strong non-polar characteristics. Both Cao (2025) and Li (2022) [14,15] had developed GC-MS and liquid chromatography/high-resolution mass spectrometry (LC-HRMS) methods to detect chlorothalonil and 4-hydroxychlorothalonil, respectively, but did not achieve their simultaneous detection. While atmospheric pressure chemical ionization (APCI) can detect chlorothalonil through gas-phase chemical ionization mechanisms, addressing ESI’s limitation for weakly polar compounds, it shows insufficient sensitivity for 4-hydroxy-chlorothalonil. Consequently, the development of an optimized method for simultaneous detection of both compounds with high accuracy and sensitivity under APCI mode represents a critical technical challenge.

The detection of chlorothalonil residues in sulfur-rich vegetables (e.g., cabbage, broccoli) presents unique analytical challenges. These vegetables contain substantial amounts of glucosinolates and their endogenous enzyme, myrosinase, which are spatially segregated within intact cells. Upon tissue disruption during sample preparation, cellular damage triggers enzymatic reactions, leading to rapid hydrolysis of glucosinolates into reactive compounds such as isothiocyanates (ITCs). These compounds can attack the chlorine atoms of chlorothalonil through nucleophilic substitution reactions, causing irreversible degradation of the target analyte and resulting in significantly underestimated detection results [14]. To inhibit the glucosinolate/myrosinase reaction, current research primarily focuses on physical deactivation methods (e.g., blanching, microwave treatment) and chemical inhibition approaches (e.g., pH adjustment, metal chelator addition) [16,17,18]. While microwave deactivation (800 W, 1 min) can rapidly inactivate the enzyme, its effectiveness is influenced by sample homogeneity, water content, and heat transfer variability, potentially leading to localized overheating or incomplete deactivation. In contrast, acid inhibition methods disrupt the enzyme’s active site through pH reduction, offering advantages of operational simplicity and cost-effectiveness. However, the inhibition efficiency of this approach in complex matrices has not been systematically evaluated.

To address the technical challenges of undetectable or significantly low recovery of chlorothalonil in sulfur-containing vegetables, this study developed a synergistic inhibition strategy involving sample pre-cooling at 4 °C and pre-soaking in citric acid solution to reduce myrosinase activity, thereby minimizing chlorothalonil degradation. Based on the use of an APCI source, we have also developed a highly sensitive UHPLC-MS/MS method for the simultaneous determination of chlorothalonil and 4-hydroxy-chlorothalonil in sulfur-rich vegetables. The proposed method provides reliable technical support for the accurate detection and risk assessment of chlorothalonil contamination in sulfur-containing vegetables.

## 2. Materials and Methods

### 2.1. Chemicals and Reagents

Analytical grade chemicals including acetone (≥99.5%), anhydrous sodium sulfate (≥99.0%), anhydrous magnesium sulfate (≥98.0%), sodium chloride (≥99.5%), and citric acid monohydrate (≥99.5%) were purchased from Sinopharm Chemical Reagent Co., Ltd. (Shanghai, China). Chromatographic grade solvents including methanol (≥99.9%), acetonitrile (≥99.9%), formic acid (≥88%), ammonium acetate (≥97%), and ethyl acetate (≥99.9%) were obtained from Thermo Fisher Scientific Inc. (Waltham, MA, USA). Chromatographic grade n-Hexane (≥95%) was supplied by Honeywell Burdick & Jackson (Muskegon, MI, USA). Graphitized carbon black (GCB, PestiCarb powder, 200–400 mesh) was purchased from Tianjin Boner-Agela Technologies Co., Ltd. (Tianjin, China).

Chlorothalonil and 4-hydroxy-chlorothalonil standard solutions, with initial concentrations of (1000 ± 5) μg/mL in acetone and acetonitrile, respectively, were obtained from Alta Scientific Co., Ltd. (Tianjin, China). All standards were stored at −20 °C and serially diluted with acetonitrile to prepare working solutions immediately before use.

### 2.2. Instrument and Equipment

The analytical system consisted of an ACQUITY UHPLC I-Class ultra-performance liquid chromatography system coupled with a Xevo TQ-S triple quadrupole mass spectrometer equipped with an atmospheric pressure chemical ionization (APCI) source and Masslynx 4.1 workstation (Waters Corporation, Milford, MA, USA). Chromatographic separation was achieved using a Poroshell 120 EC-C18 column (4.6 mm × 100 mm, 2.5 μm; Agilent Technologies, Santa Clara, CA, USA).

Sample preparation was performed using an IKA MS3 vortex mixer (IKA Werke GmbH & Co. KG, Staufen, Germany) and a Milli-Q A10 ultrapure water system (Millipore Corporation, Burlington, MA, USA). Sample homogenization was conducted using a CBS2066E blender (Airmate Electrical Appliances (Shenzhen Co., Ltd., Shenzhen, China), and centrifugation was performed using an H1850 centrifuge (Hunan Xiangyi Laboratory Instrument Development Co., Ltd., Changsha, China).

### 2.3. Sample Collection, Preparation, and Pretreatment

Blank samples of cabbage, broccoli, and radish were obtained from the Jiyang experimental base (Jinan, China), where they were cultivated without chlorothalonil application and served as matrix blanks for method development. Fifteen additional market samples were randomly collected from local markets in Jinan to ensure sample representativeness for method validation. All samples and extraction reagents were stored at 4 °C to prevent chlorothalonil degradation prior to analysis.

**Figure 1:**
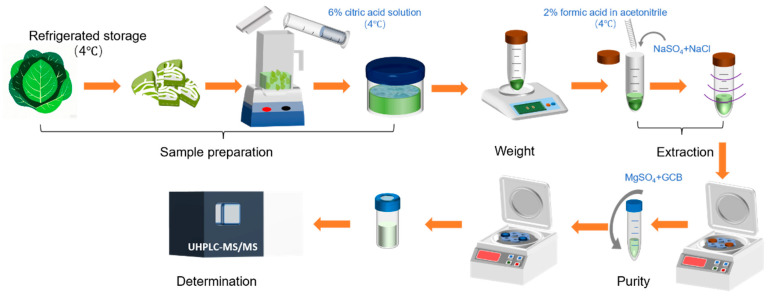
Sample preparation and pretreatment process diagram. Purple lines: vortex oscillation; grey arrows: the substances were added.

The refrigerated samples were removed and cut into 1–2 cm pieces, as shown in Figure 1. The pre-cut samples were then placed in a blender and immersed in 200 mL of refrigerated 6% citric acid solution for 5 min. Subsequently, the samples were homogenized to ensure thorough mixing. The homogenized samples were stored under refrigeration or in a frozen state until further analysis.

The sample pretreatment procedure is illustrated in Figure 1. Briefly, 10.0 ± 0.1 g of refrigerated sample was weighed into a 50 mL polypropylene centrifuge tube. Subsequently, 10 mL of refrigerated 2% formic acid/acetonitrile solution was added, and the mixture was vortexed for 1 min. Following this, 1.5 g of sodium chloride and 6 g of anhydrous sodium sulfate were sequentially added, immediately shaken to disperse, and vigorously vortexed for 1 min, and followed by refrigerated centrifugation (5000× *g*, 5 min, 4 °C). A 5 mL aliquot of the supernatant was transferred to a 10 mL centrifuge tube, to which 900 mg of magnesium sulfate and 10 mg of graphitized carbon black (GCB) adsorbent were added [19]. After vortex shock and refrigerated centrifugation (5000× *g*, 5 min, 4 °C), the extract was filtered through a 0.22 μm membrane filter (Agilent Technologies) prior to UHPLC-MS/MS analysis.

### 2.4. Enzyme Inhibition Strategy

To mitigate enzymatic reactions during sample pretreatment and reduce the degradation of chlorothalonil in sulfur-rich vegetables, thereby ensuring accurate detection, this study investigated the effects of low-temperature and acidification strategies. Then, 200 g of cabbage samples, confirmed to be free of chlorothalonil residues, was cut into 1 cm pieces and spiked with chlorothalonil and 4-hydroxy-chlorothalonil at a concentration of 0.1 mg/kg. The recoveries were determined using four sample storage and preparation approaches.

Furthermore, this study investigated the impact of acidified extraction solvents on detection outcomes during sample pretreatment. The extraction efficiency, expressed as recoveries, of chlorothalonil and 4-hydroxy-chlorothalonil was compared using different solvent systems, including acetonitrile, 1% formic acid/acetonitrile, 2% formic acid/acetonitrile, and 3% formic acid/acetonitrile.

### 2.5. UHPLC-MS/MS Analysis

Chromatographic separation was performed under the following conditions: column temperature maintained at 40 °C, autosampler temperature set at 4 °C, and injection volume of 2.0 μL. The mobile phase consisted of 2 mM ammonium acetate in water (phase A) and acetonitrile (phase B) at a flow rate of 0.7 mL/min. The gradient elution program was optimized as follows: 0–0.5 min, 80% A; 0.5–4 min, 80% A to 20% A; 4–6 min, 20% A; 6–7 min, 20% A to 80% A; 7–9 min, 80% A.

Mass spectrometric detection was conducted using atmospheric pressure chemical ionization in negative ion mode (APCI^−^). Multiple reaction monitoring (MRM) was employed with the following parameters: capillary voltage at 3.0 kV, cone voltage at 30 V, ion source temperature at 150 °C, cone gas flow at 150 L/h, desolvation gas flow at 1000 L/h, APCI probe temperature at 600 °C, and collision cell pressure maintained at 7.58 × 10^−4^ mbar. The optimized collision energies and ion transition parameters are detailed in Table 1.

### 2.6. Method Validation

To evaluate the applicability of the proposed method, key parameters including limit of detection (LOD), limit of quantification (LOQ), linearity, matrix effect, accuracy, and precision were systematically assessed. Chlorothalonil and 4-hydroxy-chlorothalonil were spiked into cabbage samples, and the concentrations corresponding to signal-to-noise ratios (S/N) of ≥3 and ≥10 were designated as the LOD and LOQ, respectively. Calibration curves were prepared in both acetonitrile and blank matrix extracts (cabbage, broccoli, and radish) at concentrations of 2, 5, 10, 20, 50, and 100 μg/L, with each concentration analyzed in triplicate. Linearity was assessed using the correlation coefficient (r) of the curves. Matrix effects were quantitatively evaluated by comparing the slopes of matrix-matched calibration curves with those of solvent-based calibration curves. The matrix effect was calculated following the formula *ME* (%) = $(\frac{slope\left(matrix\right)}{slope\left(reference\right)}-1)\times 100$. A matrix effect was categorized as weak when the absolute value of ME was ≤20%, moderate when it ranged from 20% to 50%, and strong when it surpassed 50%.

To evaluate the accuracy and precision of the proposed method, representative blank samples of different categories, including cabbage, broccoli, and radish, were selected for spiked recovery experiments. The spiked concentrations were 0.01, 0.05, and 0.2 mg/kg, respectively, with six replicates at each level. Both intra-day and inter-day precision were determined through replicate analyses. The intra-day precision was assessed by consecutive measurements within a single analytical run, while the inter-day precision was evaluated through parallel determinations conducted after 1-day and 3-day intervals.

## 3. Results and Discussion

### 3.1. Optimization of Mass Spectrum Conditions

Chlorothalonil, characterized by its weak polarity, exhibits poor ionization efficiency in electrospray ionization (ESI) due to the difficulty in forming stable charged droplets. To achieve high-sensitivity detection, this study employed APCI as an alternative to ESI, significantly enhancing ionization efficiency and detection specificity [20].

**Figure 2:**
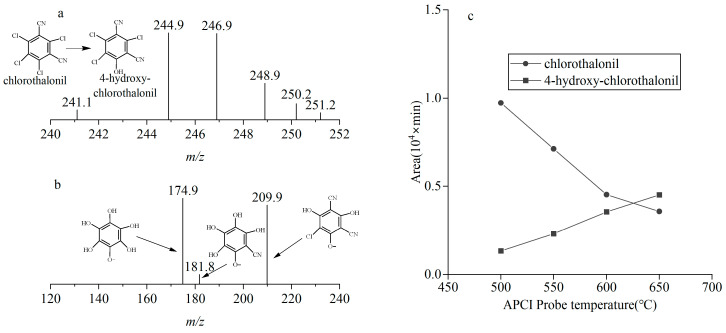
Mass spectrum of chlorothalonil and 4-hydroxy-chlorothalonil by APCI negative ionization: (**a**) the precursor ions, *m*/*z* 240~*m*/*z* 252; (**b**) the prominent ions, *m*/*z* 120~*m*/*z* 240; (**c**) relationship between APCI probe temperature and signal intensity. In (**a**), the arrows indicate the conversion of chlorothalonil to 4-hydroxychlorothalonil, while in (**b**), the arrows represent the compound structures corresponding to the characteristic ions.

As shown in Figure 2a, under APCI negative ion mode, chlorothalonil and 4-hydroxy-chlorothalonil demonstrated similar primary mass spectra, including comparable precursor ion abundances and fragmentation patterns. Notably, the ions at *m*/*z* 244.9 and *m*/*z* 246.9 showed consistent and high relative abundances, followed by *m*/*z* 248.9. The isotopic peak cluster intensity ratio of 3:3:1 aligns with the theoretical distribution for molecules containing three chlorine atoms, confirming the presence of three chlorine atoms in the precursor ions. Based on these observations, the precursor ions of 4-hydroxy-chlorothalonil and chlorothalonil were identified as [M-H]^−^ and [M-Cl+O]^−^, respectively. This ionization behavior is consistent with that observed in atmospheric pressure photoionization (APPI) [21]. The formation of the [M-Cl+O]^−^ peak for chlorothalonil can be attributed to a nucleophilic substitution reaction induced by the corona discharge needle in APCI, where the chlorine atom at the 4-position is replaced by a hydroxyl group. For both chlorothalonil and 4-hydroxy-chlorothalonil, the precursor ion at *m*/*z* 244.9, which exhibited the highest abundance, was selected for further analysis. Under collision-induced dissociation (CID) mode, secondary mass spectrometry scanning revealed three prominent ions at *m*/*z* 174.9, *m*/*z* 209.9, and *m*/*z* 181.8, as illustrated in Figure 2b. These characteristic ions are attributed to dechlorination and hydroxyl substitution on the benzene ring of chlorothalonil, a fragmentation pattern consistent with that observed in ESI mode [22]. Based on the fragmentation patterns and ion abundances of chlorothalonil and 4-hydroxy-chlorothalonil, *m*/*z* 244.9 → 174.9 was selected as the quantitative ion for both compounds, while *m*/*z* 244.9 → 209.9 and *m*/*z* 244.9 → 181.8 were qualitative ions.

To address the challenges associated with the strong polarity and low gasification efficiency of 4-hydroxy-chlorothalonil, the APCI probe temperature was optimized to enhance the signal response. As illustrated in Figure 2c, when the temperature was increased from 500 °C to 650 °C, the peak area of 4-hydroxy-chlorothalonil improved approximately threefold, attributed to the breakdown of hydrogen bonds facilitating vaporization. In contrast, the peak area of chlorothalonil gradually decreased to about one-third of its initial value, likely due to accelerated thermal decomposition at higher temperatures. To balance the sensitivity of both compounds, an optimal APCI probe temperature of 600 °C was selected. At this temperature, the peak area of chlorothalonil was slightly higher than that of 4-hydroxy-chlorothalonil for the same concentration.

### 3.2. UHPLC Separation

To enhance the separation of chlorothalonil and 4-hydroxy-chlorothalonil and improve ionization efficiency in the APCI source, the performance of two C18 columns was evaluated: a Poroshell 120 EC-C18 column (4.6 mm × 100 mm, 2.5 μm; Agilent Technologies, USA) and an Alphasil VC-C18 column (2.1 mm × 100 mm, 1.9 μm; Acchrom Technologies Co., Ltd., China). Compared to the Alphasil column, the Poroshell 120 column achieves satisfactory resolution and higher mass spectrometry response. The larger particle size allows for a higher flow rate (0.7 mL/min), providing sufficient solvent molecules for the APCI source and significantly improving ionization efficiency. Consequently, the Poroshell 120 column was selected for this method.

**Figure 3:**
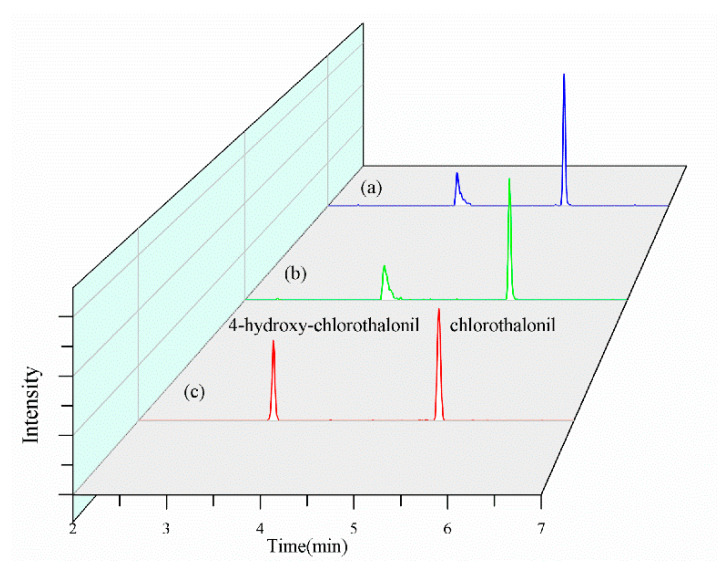
Total ion chromatogram of chlorothalonil and 4-hydroxy-chlorothalonil in (**a**) 0.1% formic acid aqueous solution; (**b**) 2 mM ammonium acetate in 0.1% formic acid aqueous solution; (**c**) 2 mM ammonium acetate aqueous solution.

This study also evaluated three mobile phase systems for chromatographic separation and mass spectrometric response: (1) 0.1% formic acid aqueous solution as mobile phase A and acetonitrile as mobile phase B; (2) 2 mM ammonium acetate in 0.1% formic acid aqueous solution as mobile phase A and acetonitrile as mobile phase B; and (3) 2 mM ammonium acetate aqueous solution as mobile phase A and acetonitrile as mobile phase B. As shown in Figure 3, the use of 2 mM ammonium acetate as mobile phase A resulted in optimal chromatographic separation. This improvement is attributed to the ion-pairing effect, which shields the activity of silanol groups, leading to better peak shapes. Additionally, this mobile phase reduced deprotonation in the APCI source, enhancing detection stability. In contrast, the presence of formic acid suppressed APCI ionization, resulting in reduced characteristic ion abundances and noticeable peak tailing when 0.1% formic acid in water or 2 mM ammonium acetate with 0.1% formic acid were used as mobile phase A. Compared to existing methods for the simultaneous detection of chlorothalonil and 4-hydroxychlorothalonil reported in the literature [21,23], our approach demonstrates significant improvements in both chromatographic resolution and peak shape characteristics.

Furthermore, the gradient program significantly influenced the characteristic ion abundances in mass spectrometry. For instance, 4-hydroxy-chlorothalonil eluted at 3.6 min with an acetonitrile proportion of 73%, while chlorothalonil eluted at 5.6 min with an acetonitrile proportion of 80%. The higher organic phase proportion accelerated desolvation, substantially enhancing ion abundances.

### 3.3. Sample Preparation and Pretreatment by Synergistic Enzyme Inhibition Strategy

Through comparative analysis of the sample preparation approaches for cabbage, the significance of enzyme inhibition in the detection of chlorothalonil in sulfur-rich vegetables was elucidated. As shown in Table 2, storage at 20 °C or 4 °C, for both samples and extraction reagents, was involved in the results comparison, as well as homogenization directly or immersed in 6% citric acid solution in advance.

The use of citric acid primarily aims to suppress its enzymatic activity by lowering the pH. To determine the optimal enzyme inhibition conditions, we evaluated the effect of different citric acid concentrations (2%, 4%, 6%, and 8%) on chlorothalonil recoveries in cabbage. The results demonstrated no significant difference in recovery between 6% and 8% citric acid treatments, but both showed markedly higher recovery compared to 2% and 4% treatments. In accordance with green chemistry principles, we selected 6% citric acid as the optimal pre-soaking solution.

**Figure 4:**
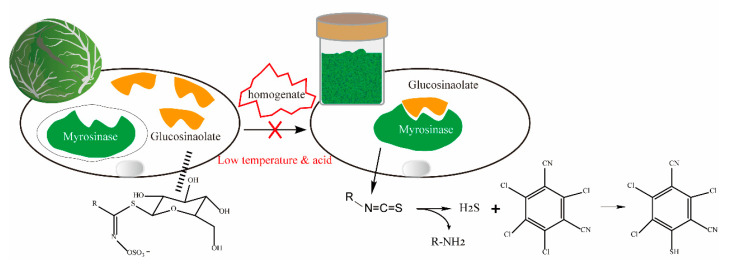
Mechanism of low-temperature and acidification synergistic enzyme inhibition strategy for the determination of chlorothalonil and 4-hydroxy-chlorothalonil.

When cabbage samples were directly homogenized at room temperature, the recovery of chlorothalonil approached 0%, indicating rapid activation of myrosinase at room temperature [24], and its enzymatic reaction products led to complete degradation of chlorothalonil, with no 4-hydroxy-chlorothalonil detected in the degradation products. Direct homogenization of cabbage samples under refrigeration resulted in a chlorothalonil recovery of 1.7%, demonstrating that refrigeration alone did not significantly mitigate chlorothalonil degradation. However, when samples were prepared with a 6% citric acid solution in advance, the chlorothalonil recovery increased to 76.5% at room temperature and 89.9% under refrigeration, respectively, underscoring the synergistic effectiveness of low temperature and acidification in enzyme inhibition. This improvement is attributed to the protonation of histidine residue (His156) at the active center of myrosinase by citric acid, as illustrated in Figure 4, which inhibits the hydrolysis of glucosinolates and prevents the conversion of ITC to amine compounds [25]. Since the optimal pH for myrosinase in sulfur-containing vegetables such as broccoli is between pH 6.5 and 7 [26], acidification of the samples plays a predominant role in enzyme inhibition. Meanwhile, low temperature acts as a secondary factor, contributing to the inhibitory effect in a synergistic manner. The differences on recovery also demonstrate that the synergistic inhibitory effect of combined low-temperature and acidification treatment is significantly more effective than that achieved by either single-factor approach alone.

It is noteworthy that the recoveries of 4-hydroxy chlorothalonil remained consistently similar across these approaches, ranging between 87.5 and 93.2%, demonstrating no significant influence from enzymatic reactions or enzyme inhibition strategies. The structure of 4-hydroxychlorothalonil features a hydroxyl group substitution at the active chlorine atom of chlorothalonil, which eliminates the nucleophilic attack site and consequently enhances its stability. This structural characteristic makes its recovery unaffected by the sample pretreatment conditions (temperature and pH) employed in the proposed method. In contrast, the stability of chlorothalonil is highly dependent on the effectiveness of enzymatic activity inhibition strategies. This study proposes a low-temperature and acidification synergistic enzyme inhibition strategy, achieving comprehensive inhibition of glucosinolate hydrolysis through dual regulation. Compared to the liquid nitrogen homogenization method for enzyme activity reduction [27], our approach offers superior operational simplicity, lower cost, and greater suitability for routine laboratory analysis.

Furthermore, this study systematically compared the extraction efficiencies of various solvent systems, including acetonitrile, 1% formic acid/acetonitrile, 2% formic acid/acetonitrile, and 3% formic acid/acetonitrile. Our findings demonstrate that the incorporation of formic acid moderately enhances the recovery of chlorothalonil in cabbage samples. Notably, the 2% formic acid/acetonitrile solution achieved optimal extraction efficiency, with a recovery of 92.4%. In contrast, the addition of formic acid showed negligible effects on the extraction of 4-hydroxy chlorothalonil, which is consistent with previous reports [11]. Taking into account multiple factors including detection cost, operational safety, and solvent durability, we selected 2% formic acid/acetonitrile as the optimal extraction solvent for subsequent analyses.

### 3.4. Method Validation Results

Sensitivity was evaluated through the limit of detection (LOD) and limit of quantitation (LOQ). Based on the signal-to-noise ratio, the LOD and LOQ for chlorothalonil and 4-hydroxy chlorothalonil in sulfur-rich vegetable samples were determined to be 0.003 mg/kg and 0.01 mg/kg, respectively.

As shown in Table 3, both chlorothalonil and 4-hydroxy-chlorothalonil exhibited good linearity within the range of 2–100 μg/L, with correlation coefficients (r) exceeding 0.99 in both acetonitrile solvent and matrix solutions (including cabbage, broccoli, and radish).

Matrix effects (MEs) were evaluated through slope ratio comparison, as ME represents a critical factor influencing the quantitative accuracy of mass spectrometry-based techniques. Co-extracted components may potentially suppress or enhance the ionization efficiency of target analytes [28]. The results indicated that chlorothalonil showed moderate suppression effects in cabbage and broccoli matrices, with ME values of 30.0% and 27.9%, respectively (20 < ME < 50), while demonstrating weak suppression in radish matrix with an ME value of 13.1% (ME < 20%). In contrast, 4-hydroxy-chlorothalonil exhibited weak suppression effects across all tested matrices, with ME values of 1.6%, 17.3%, and 2.3% in cabbage, broccoli, and radish, respectively. To enhance the accuracy of detection results, matrix-matched calibration curves were employed for quantitative analysis.

According to the results of the spiked recovery experiments, as shown in Table 4, the recovery levels of chlorothalonil and 4-hydroxy-chlorothalonil in cabbage, broccoli, and radish substrates were basically similar. The average recoveries for chlorothalonil ranged from 76.5% to 91.1%, with relative standard deviations (RSDs) below 8.1%. For 4-hydroxy chlorothalonil, the average recoveries were between 87.6% and 96.7%, with RSDs below 9.4%. The RSDs of intra-day precision of chlorothalonil and 4-hydroxy-chlorothalonil were determined as 2.6–6.7% and 1.9–7.5%, respectively, while the RSDs of inter-day precision were 3.8–7.4% and 2.4–7.6%, respectively. The accuracy and precision of the method met the requirements of SANTE/11312/2021 guidelines [29], indicating its suitability for the analysis of actual samples.

### 3.5. Analysis of Actual Samples

Fifteen sulfur-rich vegetable samples, including cabbage, broccoli, and radish, were collected and analyzed using the established method. The results revealed the presence of chlorothalonil in two cabbage samples, with residue levels of 1.5 mg/kg and 3.6 mg/kg. These values are below the MRL of 5 mg/kg set for Brassica vegetables in China. However, these residue levels still exceed the EU’s MRL of 0.01 mg/kg for chlorothalonil in vegetables (Regulation (EU) 2021/155). Such contaminated vegetables would pose significant trade risks if intended for export to EU markets. The successful detection of chlorothalonil in these sulfur-rich vegetables demonstrates the practical applicability of the proposed method and its suitability for meeting the requirements of agricultural product quality and safety monitoring.

## 4. Conclusions

This study developed a UHPLC-MS/MS method for the simultaneous determination of chlorothalonil and 4-hydroxy-chlorothalonil in sulfur-rich vegetables. By employing an APCI source instead of the conventional ESI source, the ionization efficiency of chlorothalonil was significantly improved. Further optimization of mass spectrometric and UHPLC conditions ensured optimal detection sensitivity and chromatographic separation. A low-temperature and acidification synergistic enzyme inhibition strategy was established to minimize loss on the recovery of chlorothalonil caused by enzymatic degradation during sample preparation. Method validation confirmed good sensitivity, linearity, accuracy, and precision, meeting the requirements of the SANTE/11312/2021 guidelines. The proposed synergistic enzyme inhibition strategy substantially improved the recovery of chlorothalonil in sulfur-rich vegetables, while matrix effects were found not to be the primary cause for that recovery loss. Practical application to samples in the local market will demonstrate the practical applicability of the proposed method and its suitability for monitoring the quality and safety of agricultural products.

## Figures and Tables

**Table 1:** MS/MS parameters for the determination of chlorothalonil and 4-hydroxy-chlorothalonil by APCI negative ionization.

Compound	Parent Ion (*m*/*z*)	Product Ions (*m*/*z*)	Cone Voltage (V)	Collision Energy (eV)	Dwell Time (ms)
chlorothalonil	244.9	174.9 *	40	36	100
		209.9	40	24	100
		181.8	40	30	100
4-hydroxy-chlorothalonil	244.9	174.9 *	40	36	100
		209.9	40	24	100
		181.8	40	30	100

* Quantification ion.

**Table 2:** Comparison of enzyme inhibition strategies in sample storage and preparation (*n* = 3).

Approach	Storage Temperature * (°C)	Homogenization Method	Recovery (%)	
			Chlorothalonil	4-Hydroxy-Chlorothalonil
A	20	directly homogenized	ND	89.6 ± 4.3
B	4	directly homogenized	1.7 ± 0.9	87.5 ± 3.9
C	20	immersed in 6% citric acid solution before homogenization	76.5 ± 8.4	92.3 ± 2.4
D	4	immersed in 6% citric acid solution before homogenization	89.9 ± 6.8	93.2 ± 5.1

* Includes both samples and extraction reagents; ND: not detected.

**Table 3:** LODs, LOQs, linearity ranges, regression equations, correlation coefficients, and matrix effects of chlorothalonil and 4-hydroxy-chlorothalonil standard curves.

Compound	Matrix	LOD (mg/kg)	LOQ (mg/kg)	Linearity Range (μg/L)	Regression Equation	Correlation Coefficient (r)	Matrix Effects (%)
chlorothalonil	Acetonitrile	0.003	0.01	2~100	y = 8.83x − 8.5	0.9966	NA
	Cabbage	0.003	0.01	2~100	y = 7.15x − 4.53	0.9917	30.0
	Broccoli	0.003	0.01	2~100	y = 7.27x − 1.97	0.9959	27.9
	Radish	0.003	0.01	2~100	y = 8.10x − 5.69	0.9970	13.1
4-hydroxy-chlorothalonil	Acetonitrile	0.003	0.01	2~100	y = 5.59x − 1.36	0.9966	NA
	Cabbage	0.003	0.01	2~100	y = 5.68x − 2.39	0.9926	1.6
	Broccoli	0.003	0.01	2~100	y = 6.56x − 6.76	0.9939	17.3
	Radish	0.003	0.01	2~100	y = 5.46x − 5.10	0.9927	2.3

LOD: the limit of detection; LOQ: the limit of quantification; NA: not available.

**Table 4:** The accuracy and precision of the method for the determination of chlorothalonil and 4-hydroxy-chlorothalonil in sulfur-rich vegetables.

Matrix	Compound	Spiked Level (mg/kg)	Recovery (%) (*n* = 6)	RSD (%) (*n* = 6)	Intra-Day RSD (%) (*n* = 6)	Inter-Day RSD (%) (*n* = 6)
Cabbage	Chlorothalonil	0.01	79.9	6.3	5.1	6.0
		0.05	82.0	7.8	5.9	6.8
		0.2	86.8	5.9	4.2	6.4
	4-hydroxy-chlorothalonil	0.01	89.9	4.9	4.1	5.3
		0.05	93.5	3.8	1.9	3.9
		0.2	96.1	6.1	5.6	7.0
Broccoli	Chlorothalonil	0.01	82.7	7.5	6.7	5.1
		0.05	89.3	5.4	4.0	3.8
		0.2	91.1	4.9	4.8	4.8
	4-hydroxy-chlorothalonil	0.01	92.3	5.3	7.5	7.6
		0.05	93.2	3.7	3.3	2.9
		0.2	96.7	2.8	4.8	2.4
Radish	Chlorothalonil	0.01	76.5	8.1	6.0	7.4
		0.05	81.2	6.9	2.6	3.8
		0.2	85.7	4.3	3.3	4.6
	4-hydroxy-chlorothalonil	0.01	87.6	9.4	6.1	7.5
		0.05	88.5	5.9	2.1	3.4
		0.2	91.8	3.8	2.9	2.8

## Data Availability

The original contributions presented in this study are included in the article. Further inquiries can be directed to the corresponding author.
